# Waking the sleeping dragon: gene expression profiling reveals adaptive strategies of the hibernating reptile Pogona vitticeps

**DOI:** 10.1186/s12864-019-5750-x

**Published:** 2019-06-06

**Authors:** Alexander Capraro, Denis O’Meally, Shafagh A. Waters, Hardip R. Patel, Arthur Georges, Paul D. Waters

**Affiliations:** 1 School of Biotechnology and Biomolecular Sciences, Faculty of Science, UNSW Sydney, Sydney, NSW 2052 Australia; 2 Institute for Applied Ecology, University of Canberra, Canberra, ACT 2601 Australia; 3 School of Women’s & Children’s Health, Faculty of Medicine, UNSW Sydney, Sydney, NSW 2052 Australia; 4 John Curtin School of Medical Research, Australian National University, Canberra, 2601 ACT Australia; 5 Present address: Center for Gene Therapy, Beckman Research Institute of the City of Hope, Duarte, CA 91010 USA

**Keywords:** Hibernation, *Pogona vitticeps*, Central bearded dragon, RNA sequencing, Proteomics, miRNA, Epigenetics, Stress response

## Abstract

**Background:**

Hibernation is a physiological state exploited by many animals exposed to prolonged adverse environmental conditions associated with winter. Large changes in metabolism and cellular function occur, with many stress response pathways modulated to tolerate physiological challenges that might otherwise be lethal. Many studies have sought to elucidate the molecular mechanisms of mammalian hibernation, but detailed analyses are lacking in reptiles. Here we examine gene expression in the Australian central bearded dragon (*Pogona vitticeps*) using mRNA-seq and label-free quantitative mass spectrometry in matched brain, heart and skeletal muscle samples from animals at late hibernation, 2 days post-arousal and 2 months post-arousal.

**Results:**

We identified differentially expressed genes in all tissues between hibernation and post-arousal time points; with 4264 differentially expressed genes in brain, 5340 differentially expressed genes in heart, and 5587 differentially expressed genes in skeletal muscle. Furthermore, we identified 2482 differentially expressed genes across all tissues. Proteomic analysis identified 743 proteins (58 differentially expressed) in brain, 535 (57 differentially expressed) in heart, and 337 (36 differentially expressed) in skeletal muscle. Tissue-specific analyses revealed enrichment of protective mechanisms in all tissues, including neuroprotective pathways in brain, cardiac hypertrophic processes in heart, and atrophy protective pathways in skeletal muscle. In all tissues stress response pathways were induced during hibernation, as well as evidence for gene expression regulation at transcription, translation and post-translation.

**Conclusions:**

These results reveal critical stress response pathways and protective mechanisms that allow for maintenance of both tissue-specific function, and survival during hibernation in the central bearded dragon. Furthermore, we provide evidence for multiple levels of gene expression regulation during hibernation, particularly enrichment of miRNA-mediated translational repression machinery; a process that would allow for rapid and energy efficient reactivation of translation from mature mRNA molecules at arousal. This study is the first molecular investigation of its kind in a hibernating reptile, and identifies strategies not yet observed in other hibernators to cope stress associated with this remarkable state of metabolic depression.

**Electronic supplementary material:**

The online version of this article (10.1186/s12864-019-5750-x) contains supplementary material, which is available to authorized users.

## Background

Hibernation is an extreme state of inactivity used among diverse animal lineages to cope with low or unpredictable food availability and unfavourable seasonal conditions during winter. Hibernation involves long periods of hypometabolism (torpor), often interrupted by shorter periods of euthermia (interbout arousal). These periods of euthermia allow animals to rewarm and replenish gene and protein products; processes that are virtually halted during torpor [1]. Two decades of molecular studies of hibernation have focused on mammals, such as bears and squirrels [2–8], and recently marsupials [9], with little consideration of hibernation in reptiles. Debate surrounds the use of the word ‘hibernation’ in reptiles, with the thought that the lack of active body temperature regulation and inconsistent use of torpor necessitates an alternative term, i.e. ‘brumation’ [10]. However, there is large variation in physiology even between hibernating mammals, notably in tenrec [11], implying that hibernation is not one specific physiological state. As such, herein ‘hibernation’ will be used to describe the state of reptilian winter dormancy. Much like in mammals, reptilian hibernation also involves radical changes in behaviour and physiology [12].

In mammals, hibernation is achieved through a complex reprogramming of biological processes that leads to a drastic reduction in basal metabolic rate, transcription and translation, oxygen consumption, heart rate, and core body temperature, and an increase in physiological stress tolerance [13]. Hibernators employ general adaptive responses across all tissue types, and exhibit a range of tissue-specific responses. For example, during hibernation neuroprotective processes are activated in the brain (reviewed in [14]), contractive strength is increased in the heart [15, 16], and atrophy is limited in skeletal muscle [7, 16].

Hibernation in mammals is governed transcriptionally via chromatin modification and DNA methylation, post-transcriptionally via microRNAs (miRNAs), and post-translationally via protein modifications such as SUMOylation (reviewed in [13]). While non-cleavage translational repression of mRNAs via miRNAs is thought to be important in mammalian hibernation, as of yet, there is no direct evidence.

Exploring mechanisms used by different species to cope with extreme conditions and stressors may yield information pertinent to human disease, such as age-associated neurodegeneration, muscle atrophy, and ischemia-reperfusion injury. Studies on non-traditional model species, which have evolved different physiological strategies to cope with extreme and variable conditions, provide this critically important perspective. The Australian central bearded dragon (*Pogona vitticeps*) is an excellent model to study reptilian hibernation because the genome is sequenced [17], and hibernation that mimics natural hibernation can be easily induced in captivity. Under natural conditions, bearded dragons hibernate by burying themselves in the soil or seeking refuge in fallen logs or tree stumps [12]. Typically, hibernation occurs between May and September, the coldest months of the year, where temperatures range from 5 °C to 18 °C. While physiological studies of bearded dragon hibernation in the wild is lacking, the nature of hibernation sites (buried) suggests the lizards have reduced breathing and heart rates during hibernation, with body temperatures reflecting that of ambient temperature (as they are ectothermic). However, unlike mammalian hibernators, the central bearded dragon is not known to have interbout arousals, with rewarming achieved through basking after arousal.

We profiled gene expression using mRNA sequencing (mRNA-seq) in three tissues (brain, heart and skeletal muscle) at three time points: 1) late hibernation; 2) 2 days post-arousal (pre-feed); and 3) 2 months post-arousal. We performed label-free proteomic quantification in the same three tissues at two time points: 1) late hibernation and 2) 2 months post-arousal. Differentially expressed genes and proteins were analyzed to determine overrepresented biological pathways during hibernating and waking periods. We discovered tissue-specific pathways that protect against the stress of reptilian hibernation and provide the evidence for multiple levels of gene expression regulation that may govern the physiological changes associated with hibernation.

## Results

### Differential gene and protein expression

**Fig. 1:**
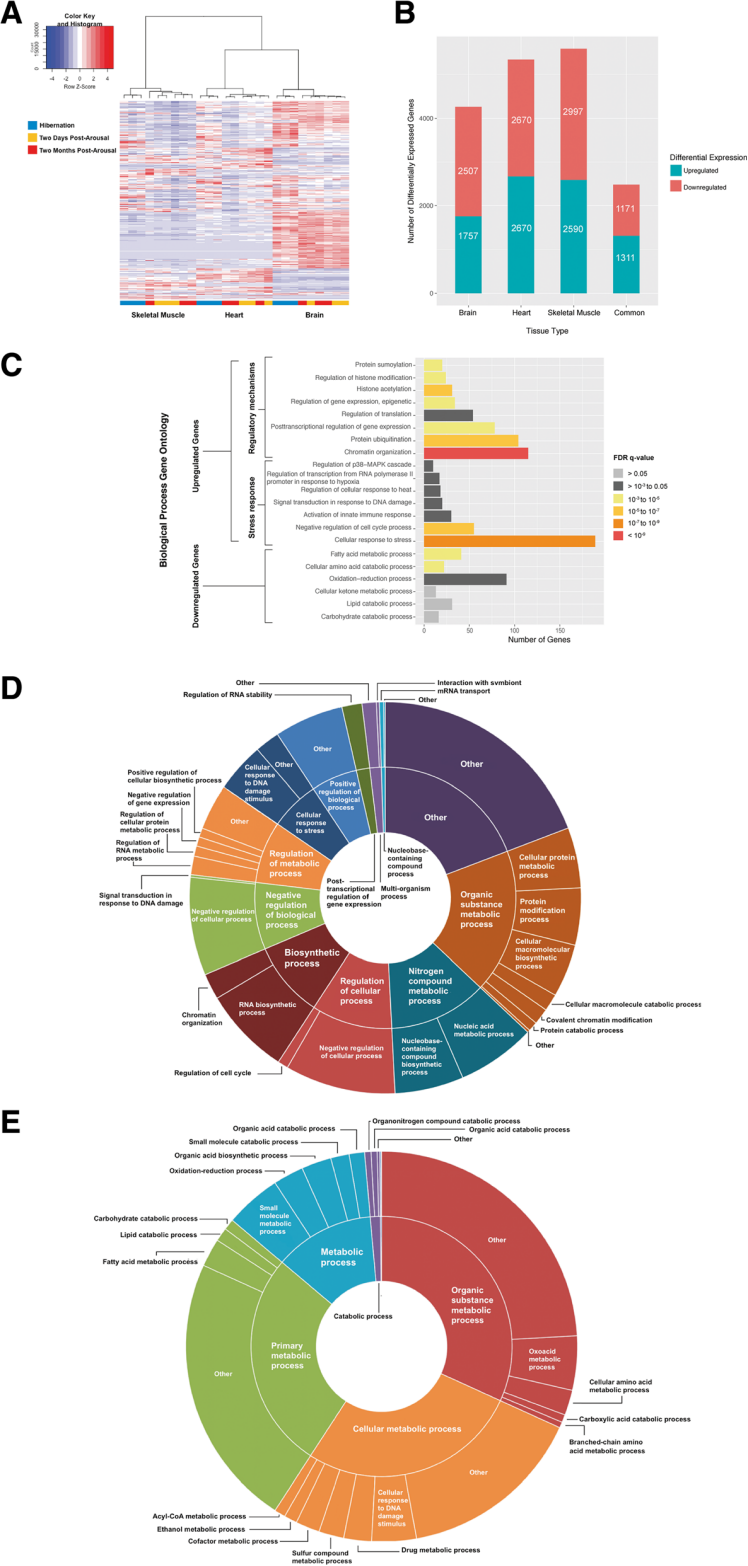
Differential gene expression and gene ontology enrichment analysis. **a** Heatmap of the 3000 most highly expressed genes in all 27 samples with hierarchical clustering of samples. Each column represents a sample, and each row represents a gene. Each tile in the heatmap shows the normalized expression of a gene (Z-score), which was calculated by subtracting the mean expression value (counts per million) of a gene across all samples from the sample specific expression value, then divided by the standard deviation of the mean expression value of the gene. Hierarchical clustering and the dendogram were calculated using Ward’s method. Colour key shows Z-score, with blue indicating lower expression and red indicating higher expression compared to the mean across all samples. **b** Bar plot of the number of differentially expressed genes during hibernation as calculated in EdgeR (Log_2_ fold change > 0.585 – i.e. 1.5-fold change – and FDR < 0.05). The number of differentially expressed genes are overlaid on the bars. **c** Bar plot of selected enriched gene ontology (GO) terms of upregulated and downregulated genes common to all tissues during hibernation, with color indicating FDR (q-value) of the GO term. **d** Donut plot of significantly enriched (FDR < 0.05) biological process GO terms for upregulated genes common to all tissues in hibernating individuals. **e** Donut plot of significantly enriched biological process GO terms for downregulated genes common to all tissues in hibernating individuals. The size of each segment is relative to the number of genes that fall within the specific gene ontology in our dataset

Hierarchical clustering of the 3000 most highly expressed genes discovered with RNA-seq in brain, heart, and skeletal muscle grouped samples of the same tissue (Fig. 1a). Within the tissue-specific clusters, three biological replicates of hibernating individuals were separated from the two post-arousal time points, which clustered together as a single group. Since the two post-arousal time points clustered together and differential gene expression analysis revealed minor differences in expression between the two post-arousal time points (Additional file 1: Table S1), they were treated as a single time point for all subsequent differential gene expression analyses. The greatest number of differentially expressed genes between hibernating and aroused animals was observed in skeletal muscle, followed by heart, and then brain (Fig. 1b, Additional file 1: Table S1). A subset of 1311 genes was upregulated in all tissue types during hibernation and 1171 genes were downregulated (Fig. 1b).

Considering the small differences in gene expression between 2 days post-arousal and 2 months post-arousal samples (Additional file 1: Table S1), 2 days post-arousal individuals were excluded from the proteomic analysis. Across all individuals (hibernators and awake) in brain, 743 proteins were identified, with the brain-specific proteins MBP, NEFM, and ATP1A2 most abundant (Additional file 2: Table S2). Twenty-seven of these proteins were upregulated, and 31 downregulated. In heart, the most abundant proteins were the muscle-specific proteins ACT, CKM, and MYH15. Of the 535 proteins identified, 29 were upregulated, and 28 downregulated during hibernation. In skeletal muscle, 337 proteins were identified, with muscle-specific proteins (CKM, TPM2, and TNNI2) the most abundant. Twenty of these proteins were upregulated, and 16 downregulated during hibernation. Overall, the correlation between mRNA and protein expression was very limited. In brain, there were 54 differentially expressed genes in the proteome that were detected in the transcriptome. However, in the transcriptome only 14 (26%) correlated with the proteome (2 upregulated and 12 downregulated during hibernation). This low correlation was mirrored in heart. Of 50 differentially expressed genes in the proteome that were detected in the trancriptome, 15 (30%) were correlated (1 upregulated and 14 downregulated during hibernation). Finally, skeletal muscle displayed the lowest correlation between proteome and transcriptome. There were 33 differentially expressed genes in the proteome, with just two (6%) correlating with the transcriptome. This low correlation may be due to the relatively low number of identified genes in the proteome (primarily high abundance proteins) compared to the transcriptome. For example, in brain 10,000 genes were identified with a counts per million (CPM) > 10, while only 735 proteins were identified. This reflects a mere 7% of transcripts that have associated proteomic data.

### Biological processes common to all tissues examined

Gene ontology (GO) enrichment analysis of the 1311 common upregulated genes in hibernators identified 259 biological process GO terms that were significantly enriched (FDR < 0.05) (Fig. 1d and Additional file 3: Table S3). These processes belonged to two major categories: 1) regulatory mechanisms of gene expression, protein translation and protein function; 2) cellular stress response and mitigation of stress severity (Fig. 1c).

### Regulation of gene expression

**Fig. 2:**
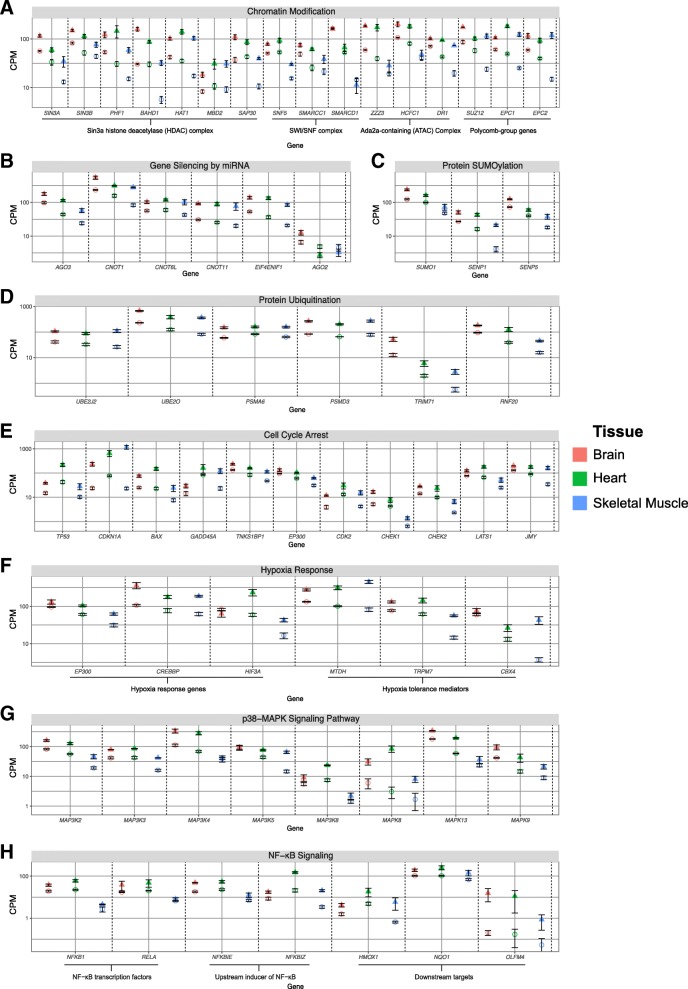
Differential expression of genes within enriched pathways during hibernation shared by all examined tissue. Mean (across biological replicates, ± 1 standard error) expression, measured in counts per million (CPM), of genes in brain (red), heart (green), and skeletal muscle (blue). Expression is shown for hibernators (triangles) and non-hibernators (circles). Panels **a** to **d** display genes related to gene expression regulatory mechanisms. Panels **e** to **h** display genes related to stress responses. The genes are from the following biological processes: **a** chromatin modification, **b** gene silencing by miRNA, **c** protein SUMOylation, **d** protein ubiquitination, **e** cell cycle arrest, **f** hypoxia response, **g** p38-MAPK signaling pathway, and **h** NF-κB signaling. All plotted on a log_10_ scale

**Fig. 3:**
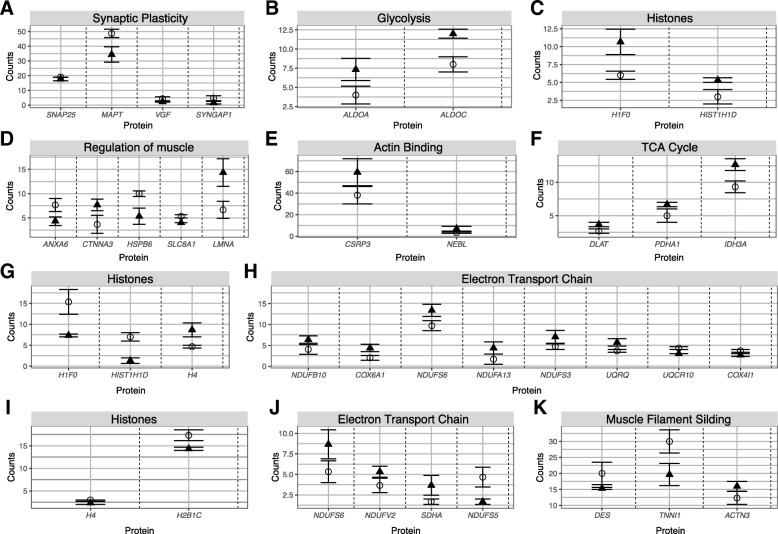
Differentially expressed proteins discovered between hibernators and 2 months post-arousal individuals within tissues. Mean (across biological replicates, ± 1 standard error) normalized spectral counts of proteins. Expression is shown for hibernators (triangles) and non-hibernators (circles). Panels **a** to **c** display differentially expressed proteins in brain; panels **d** to **h** display differentially expressed proteins in heart; and panels **i** to **k** display differentially expressed proteins in skeletal muscle. See Additional file 3: Table S3 for fold-changes and full list of protein counts

During hibernation, gene expression appears to be regulated at transcription, post-transcription, and post-translation across all tissues examined. Chromatin organization (GO:0006325) was enriched during hibernation (Fig. 1c and Additional file 3: Table S3); with 115 upregulated genes that included members of epigenetic modifying complexes such as the Sin3a histone deacetylase (HDAC) complex, the SWI/SNF complex, the Ada2a-containing (ATAC) complex, and Polycomb-group genes (Fig. 2a). Gene set enrichment analysis (GSEA) supported this finding, with enrichment of similar biological pathways, including chromatin organization (M13550) and regulation of gene expression, epigenetic (M16267), observed in during hibernation in all tissues (Additional file 4: Table S4). While chromatin modifying proteins were not differentially expressed in the proteomic data, changes in histone protein expression were evident across all tissues. H1F0 and HIST1H1D were upregulated during hibernation in brain (Fig. 3c). In contrast, these two proteins were downregulated during hibernation in heart, whereas H4 was upregulated (Fig. 3g). In skeletal muscle, H4 and H2B1C were both downregulated during hibernation (Fig. 3i).

Regulation of translation (GO:0006417) (51 genes) was enriched during hibernation, and there were 18 genes upregulated during hibernation that modulate gene expression by miRNAs (GO:0060964) (Additional file 3: Table S3). GSEA reinforced this enrichment (gene silencing by RNA; M16422) in all tissues (Additional file 4: Table S4). Importantly, these included genes required for miRNA-mediated translational repression (Fig. 2b). Notably, the cleavage-competent RNA-induced silencing complex (RISC) subunit *AGO2* (which results in mRNA degradation) was not differentially expressed (false discovery rate (FDR) > 0.05) between hibernators and non-hibernators. Finally, during hibernation there were enrichments for both protein SUMOylation (GO:0016925) (20 genes) and ubiquitination (GO:0016567) (104 genes) (Figs. 2c and d; Additional file 3: Table S3); an observation also supported by the GSEA in all tissues (Additional file 4: Table S4).

### Response to stress

Genes associated with oxidative stress, hypoxia, DNA damage and heat shock pathways were upregulated during hibernation in all examined tissues (Fig. 1c), along with 55 genes associated with negative regulation of cell cycle processes (GO:0010948) (Additional file 3: Table S3). Eleven of these 55 genes are important in the regulation of p53-mediated cell cycle arrest (Fig. 2e), including *TP53* and *CDKN1A*. Additionally, the p53-dependant G1 DNA damage response (M770) reactome pathway was highly enriched in all three tissues during hibernation (Additional file 4: Table S4). Genes that regulate transcription in response to hypoxia (GO:0061418) were also upregulated, and included the critical hypoxia response genes *EP300*, *CREBBP*, and *HIF3A*. A further three genes important for mediating hypoxia tolerance (*MTDH*, *TRPM7*, and *CBX4*) were also upregulated (Fig. 2f).

The p38 mitogen activated protein kinase (MAPK) signaling cascade is responsive to various environmental stressors [18]. Ten genes within this signaling cascade (GO:1900744), including three MAP 3Ks (MAP kinase kinase kinase), were upregulated in all tissues during hibernation. A further two MAP 3Ks and three MAPKs were also upregulated in tissues of hibernators (Fig. 2g).

Although undetected in the GO enrichment analyses, GSEA revealed that NF-κB signaling (M13738) was enriched during hibernation in all tissues (Additional file 4: Table S4). Specifically, seven genes within the NF-κB signaling pathway; a central regulator of oxidative stress response [19], were upregulated during hibernation. These included NF-κB transcription factors, upstream inducers of NF-κB signaling, and downstream target genes known to alleviate oxidative stress (Fig. 2h).

### Modulation of metabolism

Modulation of metabolic genes is a common feature in hibernators [20–27]. GO analysis of the 1171 common downregulated genes revealed 44 enriched biological processes (Fig. 1e and Additional file 1: Table S1). This observation is directly supported by the GSEA, where the majority of enriched biological pathways post-arousal were related to metabolism (Additional file 4: Table S4). Enriched biological processes were predominantly related to metabolism, including: lipid catabolic processes (31 genes – GO:0016042), oxidation-reduction processes (91 genes – GO:0055114), and carbohydrate catabolic processes (16 genes – GO:0016052) (Additional file 3: Table S3). Furthermore, we observed a downregulation of three key ketone metabolic genes (*BDH2*, *ACAT1*, and *OXCT1*), which are necessary for metabolism when liver glycogen is depleted (Additional file 1: Table S1). Downregulated carbohydrate catabolism genes were predominantly related to glycoprotein and glycolipid metabolism (*NEU1*, *NEU2*, *ENOSF1*, and *NAGA*), glycosyl metabolism (*AGL* and *MAN2C1*), and galactose metabolism (*GALE* and *GALT*) (Additional file 1: Table S1).

Enriched GO terms of common upregulated genes during hibernation related to metabolism were predominantly regarded carbohydrate metabolism, including regulation of carbohydrate metabolic process (29 genes – GO:0006109) and regulation of gluconeogenesis (11 genes – GO:0006111). Specifically, this included *PFKFB3*, which stimulates glycolysis, *GSK3A*, which controls glycogen synthesis, and *FBP1*, the rate limiting enzyme of gluconeogenesis.

Corroborating our RNA-seq results, the proteomic analysis revealed differential expression of proteins involved in metabolic processes (particularly glucose metabolism) during hibernation in all tissues. In brain, two proteins upregulated during hibernation (ALDOA and ALDOC) are critical enzymes in glycolysis (Fig. 3b). In heart, six upregulated proteins (NDUFB10, COX6A1, NDUFS6, NDUFA13, NDUFS3, and UQRQ), and two downregulated proteins (UQCR10 and COX4I1) are important in the respiratory electron transport chain. Additionally, three upregulated proteins (DLAT, PDHA1, and IDH3A) are involved in the tricarboxylic acid (TCA) cycle. In skeletal muscle three proteins (NDUFV2, NDUFS6, and SDHA), important in the respiratory electron transport chain, were also upregulated during hibernation, whereas one (NDUFA5) was downregulated.

### Tissue-specific responses during hibernation

**Fig. 4:**
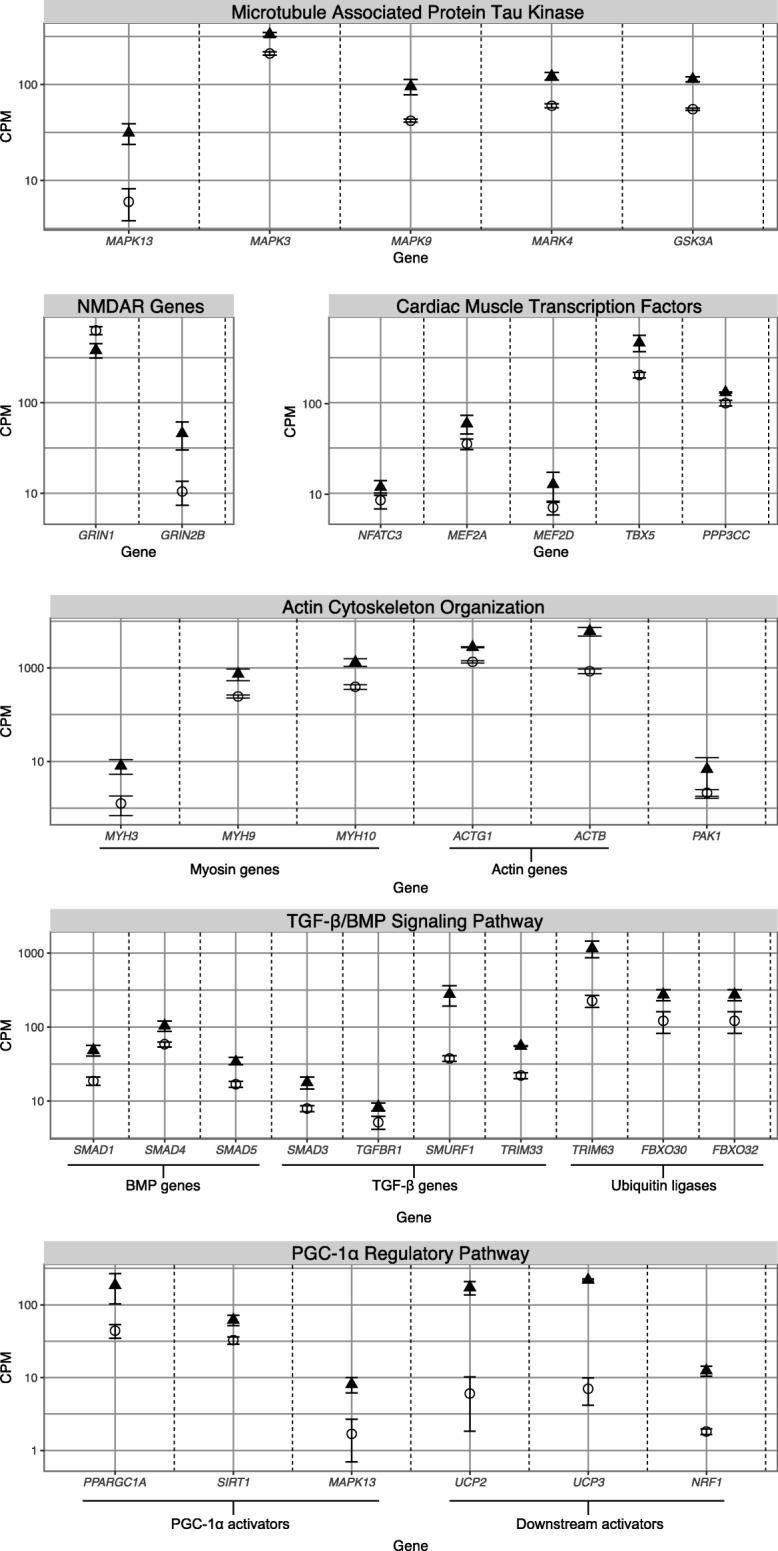
Differential expression of genes within tissue-specific processes. Mean expression (across biological replicates, ± 1 standard error), measured in counts per million (CPM). Expression is shown for hibernators (triangles) and non-hibernators (circles). The genes are from the following brain-specific processes: **a** microtubule-associated protein Tau kinases, **b** N-methyl-D-aspartate receptor genes; heart-specific processes: **c** actin cytoskeleton organization, **d** cardiac muscle transcription factors; and skeletal muscle-specific processes: **e** the TGF-β signaling pathway/BMP pathway, and **f** the PGC-1α pathway. See Additional file 1: Table S1 for fold-changes and false discovery rates

GSEA revealed an enrichment for the Kyoto Encyclopedia of Genes and Genomes (KEGG) disease pathway Alzheimer’s disease (H00056) in the brain post-arousal (Additional file 4: Table S4). Three genes downregulated during hibernation (*PS1*, *PS2*, and *APOE*) are linked to early onset Alzheimer’s disease (Additional file 1: Table S1). Furthermore, five genes responsible for the phosphorylation of the microtubule associated protein tau (MAPT – an important protein in the central nervous system) were upregulated during hibernation (Fig. 4a). Additionally, in the bearded dragon, we observed that two N-methyl-D-aspartate receptor (NMDAR) genes were differentially expressed during hibernation: *GRIN1* was downregulated during hibernation, and *GRIN2B* was upregulated during hibernation (Fig. 4b). Proteomic analysis revealed four proteins involved in synaptic plasticity (SNAP25, MAPT, VGF, and SYNGAP1) were downregulated in brain during hibernation (Fig. 3a).

During mammalian hibernation, transcription factors responsible for cardiac muscle development, and induction of cardiac hypertrophy, are important in maintaining cardiac function [28]. In bearded dragon, four cardiac transcription factors and *PPP3CC* (a cardiac hypertrophy regulator) were upregulated in heart during hibernation (Fig. 4c). Furthermore, genes required for cardiac remodeling and associated with actin cytoskeleton modulation were upregulated during hibernation, including three myosin genes and two actin genes (Fig. 4d). Proteomic analysis revealed 5 differentially expressed proteins (ANXA6, CTNNA3, HSPB6, SLC8A1, and LMNA) involved in regulating muscle system processes (Fig. 3d), and 2 proteins (CSRP3 and NEBL) involved in actin binding (Fig. 3e).

In skeletal muscle, the transforming growth factor beta-receptor (TGF-β) signaling pathway and bone morphogenetic protein (BMP) signaling pathway act antagonistically to balance muscle atrophy and hypertrophy [29]. We observed upregulation of three positive regulators of the BMP signaling pathway, and four positive regulators of the TGF-β pathway. Three muscle atrophy-related ubiquitin ligases were also upregulated during hibernation (Fig. 4e). The critical TGF-β gene myostatin (*MSTN*) was not differentially expressed between hibernating and awake animals. Finally, we observed upregulation of *PPARGCIA* (which encodes PGC-1α; the master regulator of mitochondrial biogenesis)*,* along with two of its activators, and three downstream targets (Fig. 4f); a process which is known to be important for prevention of muscle atrophy in mammals [30]. Three differentially expressed proteins identified by proteomic analysis are involved in muscle function (DES, TNNI1, and ACTN3); particularly muscle filament sliding (Fig. 3k).

## Discussion

Hibernation in reptiles is poorly studied compared to mammals. Beyond large-scale physiological responses, such as reduced heart and metabolic rate [31], the strategies common (and different) to hibernators from the two clades remain largely unknown. This study is the first to provide insight into the molecular pathways employed by a reptile during hibernation. We identified similarities between mammal and reptile hibernation, as well as responses that may be novel to the bearded dragon.

Recently, the use of steady state abundances of mRNA and proteins during hibernation has been scrutinized given that hibernation is a non-steady state condition. Some protiens do not function during hibernation as they do in steady state conditions, notably regulation of transcription by p53 [32]. However, for this study, we assume functional equivalence of biological processes during hibernation and after arousal. Additionally, transcriptomic and proteomic profiles were correlated to gain a multi-level understanding. Biological pathways (identified by gene ontology and gene set enrichment analyses) and downstream targets, rather than specific genes, were focused on to gain a more nuanced representation of physiological responses during hibernation.

### Control of gene expression

**Fig. 5:**
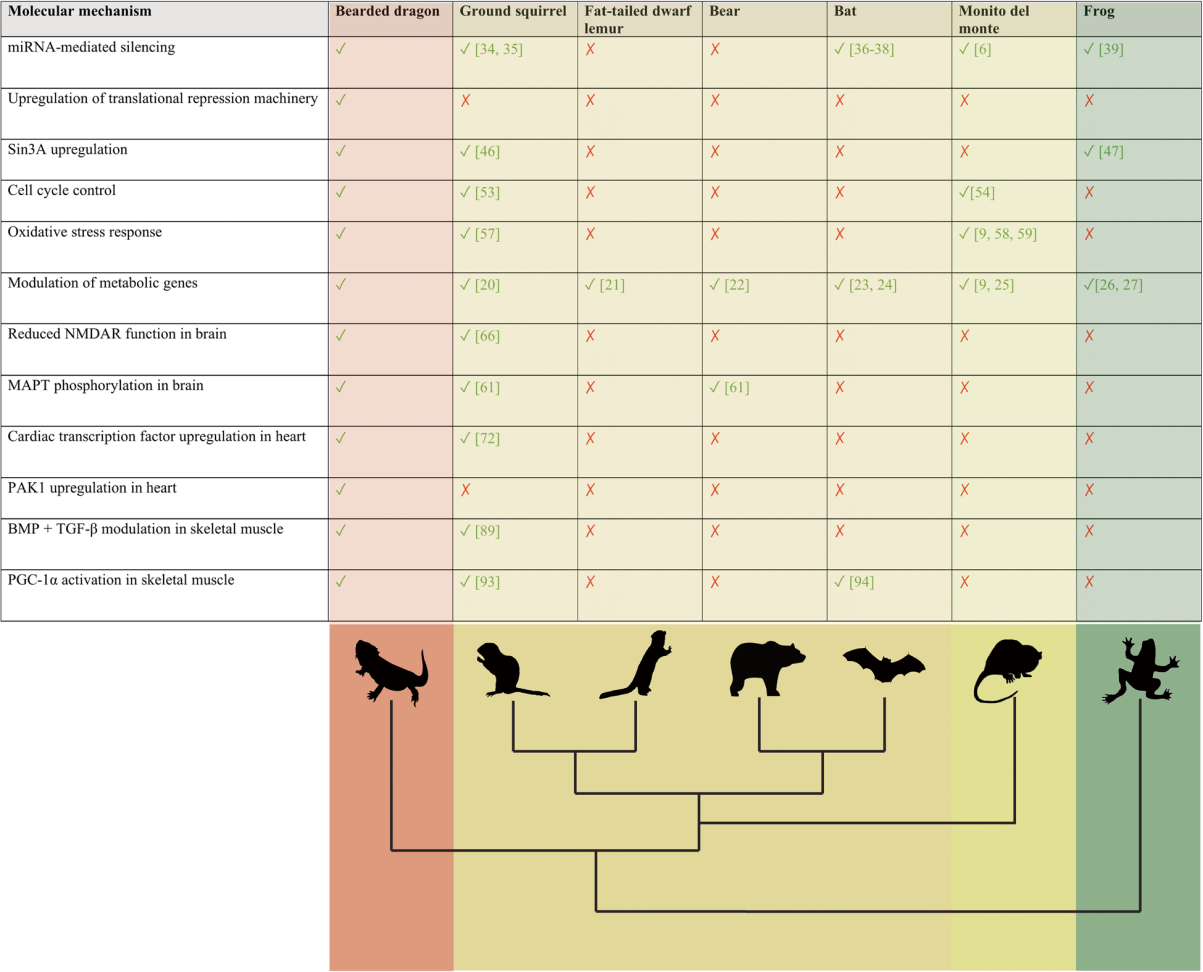
Summary of molecular mechanisms utilized during hibernation by the central bearded dragon and other hibernators. For each biological process, a tick indicates there is sufficient evidence to suggest that the process occurs, whereas a cross indicates a lack of, or insufficient, evidence that the process occurs rather than not occurring. Major vertebrate clades are identified by different colors (pink – reptile, yellow – mammal, and green – amphibian) on the phylogeny. All images used are the author’s own work

Evidence for multiple levels of gene regulation in hibernating bearded dragons was observed, which is unsurprising considering that alteration of gene expression is known to be critical for hibernation in mammals [13]. Accordingly, we identified upregulation of genes involved in RISC-mediated gene silencing in all tissues of hibernating bearded dragons (Fig. 2b). Tissue-specific miRNA expression has been reported during hibernation in thirteen-lined ground squirrels [33–35], little brown bats (*Myotis lucifugus*) [36–38], monito del monte (*Dromiciops gliroides*) [6], and wood frog (*Rana sylvatica*) [39] (Fig. 5).

The CCR4-NOT complex is a master regulator of gene expression and is required for RISC-mediated translational repression via the recruitment of the translation initiation factor 4E-T [40–43]. During hibernation, we identified upregulation of three key CCR4-NOT complex subunits, in addition to *EIF4ENIF1*, which encodes for 4E-T. Furthermore, in hibernating animals *AGO2* (necessary for cleavage-competent RISC) was not upregulated, instead *AGO3*; the catalytic subunit of non-cleavage-competent RISCs [44], was upregulated (Fig. 2b). We propose that an enrichment of non-cleavage-competent RISC machinery during hibernation may represent an important gene regulatory pathway for bearded dragon hibernation.

Together with enhanced stability and polyadenylation of mRNAs [45], non-cleavage miRNA-mediated repression of mRNAs would allow for energy efficient regulation of gene expression. Upon arousal from hibernation reactivation of translation from mature mRNA molecules does not require immediate transcription and RNA processing. This would explain how bearded dragons are able to restore normal metabolic function promptly after arousal; such as if they are disturbed during hibernation, and after emergence from hibernation.

Restructuring of chromatin appears to be necessary for gene expression regulation during vertebrate hibernation. Increased expression of components of the Sin3A-HDAC complex is common to bearded dragons (Fig. 2a), hibernating squirrels and frogs [46–49] (Fig. 5). Increased expression of genes in the silencing complexes ATAC and SWI/SNF, along with Polycomb group proteins (Fig. 2a), suggests that transcriptional control during hibernation requires the complicated interaction of many epigenetic modifiers. While differentially expressed chromatin remodeling proteins were not detected in the proteomic data, differential histone expression between hibernators and non-hibernators was apparent in all tissues (Fig. 3c, g and i). The role of histone expression in phenotypic plasticity is largely unknown; however, histone expression is important in genomic stability [50, 51], suggesting a potentially important role in hibernation.

An increased expression of small ubiquitin-like modifiers (SUMO) and ubiquitination genes was also observed during hibernation (Figs. 2c and d), which are known to modulate protein function in mammalian hibernation (reviewed in [13]). Given the important role SUMOylation plays in cellular stress protection (reviewed in [52]), reversible post-translational regulation appears to be a universal mechanism involved in vertebrate hibernation and stress response.

### Response to cellular stress

Cellular stress responses in the bearded dragon appear consistent with those of mammalian hibernators [53, 54](Fig. 5). Patterns of cell cycle arrest are a common observation during hibernation in thirteen-lined ground squirrels and in hypoxic red-eared slider turtles [53, 55] (Fig. 5). In bearded dragon, the p53 stress response pathway may be important in mediating this response. During hibernation in ground squirrels, Pan et al. demonstrated that while p53 localizes to the nucleus, recruits RNA polymerase II and binds DNA, the lack of target gene activation suggests that p53 does not function equivalently during hibernation [32]. However, in bearded dragons, critical target genes (including *CDKN1A*, *BAX*, and *GADD45A*) were transcriptionally upregulated during hibernation in all tissues (Fig. 2e), suggesting that p53 is indeed functional. In bearded dragons, the oxidative stress response may be guided by the NF-κB stress response pathway; much like in mammalian hibernators [56–59] (Fig. 5). Increased expression of key target genes that protect against oxidative damage (*HMOX1*, *NQO1*, and *OLFM4*) during hibernation suggests active protection from the sudden upsurge of reactive oxygen species that follows metabolic arousal from hibernation.

Our data implies that increased neuroprotection in brain during hibernation in bearded dragons may be governed by reduced N-methyl-D-aspartate receptor (NMDAR) function, ultimately preventing excitotoxicity: neuronal death by over-activation of glutamate receptors [60]. Downregulation of the NMDAR NR1 subunit gene *GRIN1* suggests lowered abundance of NMDARs at synapses, thus reducing capacity for excitotoxicity (Fig. 4b).

Increased expression of MAPT kinases in the brain could result in hyperphosphoryation of MAPT (Fig. 4a); a process that occurs in some hibernating mammals [61](Fig. 5) and is rapidly reversed upon arousal [62–64]. Phosphorylated MAPT has reduced affinity for microtubules, which is suggested to cause disruption of NMDAR anchoring and, therefore, neuroprotection (reviewed in [65]). This process is proposed to protect against excitotoxicity in hibernating ground squirrels [66, 67], anoxia-tolerant turtles [66, 67], and hypoxia and ischemia tolerance in the brains of rats and piglets [68–70]. Notably, MAPT-deficient mice are protected from excitotoxic brain damage [71]. In bearded dragons, *MAPT* mRNA expression did not vary significantly between hibernating and post-arousal time points, however, protein expression did (Fig. 3a). The decreased abundance of MAPT protein in hibernating bearded dragons suggests that excitotoxicity prevention (via reduced receptor abundance and stability) is a critical protective measure in the brains of hibernators.

During mammal hibernation, cardiac hypertrophy increases contractile strength [15, 72]. Unlike hypertrophic cardiomyopathy disease in humans, cardiac hypertrophy in hibernators is beneficial and quickly reversed upon arousal [72]. The cardiac-specific transcription factors with increased expression during bearded dragon hibernation (Fig. 4c) have function in promoting cardiac hypertrophy [73], cardiac-specific gene expression [74], cardiac remodeling [75], and proper heart development [76]. Our data reflect those from hibernating ground squirrels (Fig. 5) [72, 77–80].

Cardiac hypertrophy requires modulation of the actin cytoskeleton and sarcomeres (the functional unit of muscle cells) [81]. This is mirrored by upregulation of actin and myosin genes during bearded dragon hibernation (Fig. 4d). Maintenance of proper cardiac function was also revealed by the higher protein abundance of CSRP3 and NEBL during hibernation (Fig. 3e). CSRP3 and NEBL bind actin and are important in maintaining muscle structure [82, 83], with mutations causing cardiomyopathy in mammals [84, 85].

Unique to bearded dragons, the important actin cytoskeletal gene *PAK1* was upregulated in heart during hibernation (Fig. 5d). PAK1 regulates excitability and contractibility of cardiomyocytes (reviewed in [86]); with over-expression improving cardiac function in mice (reviewed in [87]), and deletion worsening hypertrophic cardiomyopathy [88]. Modulating actin organization and structure appears crucial for protecting cardiac function during hibernation.

In hibernating individuials we observed upregulated pathways involved in prevention of skeletal muscle atrophy. The transforming growth factor beta (TGF-β) and bone morphogenetic protein (BMP) signaling pathways are antagonistic; they act to induce skeletal muscle atrophy and hypertrophy, respectively [29]. The increased expression of positive regulators of both pathways during hibernation is counterintuitive (Fig. 4e). However, both pathways modulate common targets (e.g. SMAD4 and the Akt/mTOR signaling cascade), and it has been suggested that normal maintenance of muscle mass results from precise regulation of both pathways [29]. In ground squirrels, members of both the TGF-β and BMP signaling pathways are also upregulated during hibernation (Fig. 5) [89].

PGC-1α (encoded by *PPARGC1A*) is critical in muscle remodeling and mitochondrial biogenesis [90, 91]. Genes that activate PGC-1α have increased expression during hibernation in bearded dragons, as do downstream targets (Fig. 4f). High levels of PGC-1α reduces muscle atrophy in non-hibernators (reviewed in [30]) by maintaining mitochondrial function, limiting inflammatory responses, and reducing ROS production and oxidative damage [92]. Induction of PGC-1α during hibernation may also mediate the switch from fast-twitch to slow-twitch muscle fibers [93, 94] (Fig. 5), which is important for protecting the muscle from fatigue post-arousal. Upregulation of genes within the PGC-1α pathway during hibernation suggests that this process is occurring in bearded dragon (Fig. 4f). Moreover, the increased abundance of proteins within the mitochondrial respiratory chain during hibernation (Fig. 3j) indicates preservation of mitochondrial function. We propose that increased expression of genes within the PGC-1α regulatory pathway contributes to resistance of skeletal muscle atrophy in hibernating animals.

## Conclusion

Here we conducted the first transcriptional profiling and proteomic analysis of a reptile during hibernation and post-arousal from hibernation. There was evidence of neuroprotective strategies in the brain, maintenance of heart function via hypertrophy, and protection against skeletal muscle atrophy via increased antioxidant capacity and mitochondrial maintenance during hibernation. Many protective strategies we observed in hibernating bearded dragons were consistent with hibernating mammals, suggesting that there are limited solutions available to tolerate such extreme stress at the cellular level. However, bearded dragons had responses not previously detected in mammals, including the enrichment of non-cleavage competent RISC machinery during hibernation.

## Methods

### Animals and tissue collection

Central bearded dragons (*Pogona vitticeps*) were captive bred and housed at the University of Canberra under a protocol approved by the University of Canberra Animal Ethics Committee (CEAE17–08) and ACT Government License to Keep (K9640). Husbandry practices fulfill the Australian Code for the Care and Use of Animals for Scientific Purposes 8th edition (2013) sections 3.2.13–3.2.23. Commercial sources of vegetables, mice and live insects (crickets and cockroaches) were provided as food, with water available ad libitum. Cages were cleaned thoroughly monthly, with superficial cleaning done daily (removal of faecal matter and unused food, maintenance of clean water containers). Logs and small branches were provided as basking perches and cardboard boxes provided as retreats. Enclosures were lit by a fluorescent lamp, a strong UVB light source, and a floodlamp (as a heat source) on a variable light:dark (L:D) cycle: August – mid-June (13hL:11hD; 22 °C), late June (2 weeks- 6hL: 18hD; 18 °C) and winter hibernation (0hL:24hD; 12 °C). For 2 weeks prior to hibernation, heat and light were reduced and animals were not fed. All heat and UV lights were turned off for 8 weeks and the facility temperature maintained at 12 °C, which stimulated any animals remaining active to hibernate. The conditions of artificial hibernation are chosen to mimic those that occur during natural hibernation, in that ambient temperatures are dropped, and light availability reduced. Body temperatures of hibernating animals was the same as ambient temperature (12 °C) due to the lack of access to heat sources. After arousal from hibernation, animals were subject to full summer conditions (13hL:11hD; 22 °C). Body temperatures of animals was at least 22 °C (ambient) with the addition of access to a heat source.

Whole brain, whole heart and femoral skeletal muscle tissue were collected from three individuals at three time points: late hibernation, 2 days post-arousal and 2 months post-arousal. All samples were used in the transcriptomic analysis, while only late hibernation and 2 months post-arousal samples were used in the proteomic analysis. All lizards were male. Tissues were collected immediately after euthanizing (lethal injection of sodium pentobarbitone 65 mg/kg by caudal venipuncture), snap frozen in liquid nitrogen and stored at − 80 °C until RNA and protein extraction. All post-arousal animals were sacrificed between zeitgeber time (ZT) 3 and ZT5, where ZT0 is lights on and ZT13 is lights off. Hibernating animals were sacrificed between circadian time (CT) 3 and CT5, where CT0 is the same time of day as ZT0, however, without lights turning on.

### RNA preparation and sequencing

Total RNA was extracted from 50 mg of each tissue. Tissue extracts were homogenized in TRIzol reagent (Thermofisher, Waltham, Massachusetts, USA) using T10 Basic ULTRA-TURRAX® Homogenizer (IKA, Staufen im Breisgau, Germany), and RNA purified using the RNeasy Mini Kit (QIAGEN, Hilden, Germany) according to the manufacturer’s instructions. An on-column DNase digestion was performed with RNase-free DNase (QIAGEN, Hilden, Germany). For each sample, 5–10 μg of high integrity RNA (RIN > 8) was poly-A selected. Libraries were constructed with the Illumina TruSeq Total RNA Stranded RNA kit, and 76 bp single-ended reads were generated on the Illumina NextSeq 500 platform at the Ramaciotti Centre for Genomics (UNSW, Australia). All sequence data have been submitted to the NCBI short read archive under the BioProject ID PRJNA476034.

Raw read quality was analyzed with FastQC (v0.11.5) [95]. Trimmomatic (v0.36) [96] was used to trim the reads to remove low quality bases with the following options: HEADCROP:12 CROP:62 SLIDINGWINDOW:4:15. Reads were mapped to the annotated reference genome of the central bearded dragon [17] with HiSat2 (v2.0.5) [97] using default options. HTseq-count (v0.9.1) [98] was used to count reads that overlapped genomic features with the following options: -s reverse -m union. Samples were normalized using the trimmed mean of M-values (TMM) method and differentially expressed genes was calculated with EdgeR (v3.20.8) [99] in a pairwise manner using the exact test method. Resultant *P*-values were adjusted using the Benjamini-Hochberg procedure to calculate FDR. Genes with a fold-change greater than 1.5 (log_2_ fold-change of 0.585) and FDR less than 0.05 were considered differentially expressed. Gene ontology enrichment analysis related to differentially expressed genes were conducted with GOrilla using the human database (GO term database last updated December 9th 2017) [100]. Unranked lists of upregulated and downregulated genes in each condition and tissues were compared to a background list. The background list only included genes that were expressed (greater than 10 counts per million) within each tissue. For differentially expressed genes common to all tissues, only genes expressed in all three tissues were included in the background list. Gene set enrichment analysis (GSEA) [101] was performed for each tissue using defaults settings. Gene sets (Collection 2: Kegg, Biocarta and Reactome; Collection 5: GO Gene sets for Biological Process, Molecular Function and Cellular Component) were downloaded from MSigDB [102]. As with the differential gene expression analysis, the two post-arousal time points were collated as a single time point. All graphs were plotted with R (3.4.2) [103], RStudio (1.1.383) [104], and ggplot2 (2.2.1) [105].

### Protein extraction and mass spectrometry

Total protein was extracted from 50 mg of tissue. Tissue extracts were homogenized in RIPA buffer (50 mM Tris-HCl pH 7.5, 1% Triton X-100, 0.5% Na-deoxycholate, 0.1% SDS, 150 mM NaCl, 2 mM EDTA), cOmplete® and EDTA-free Protease Inhibitor Cocktail (Roche, Basel, Switzerland) using T10 Basic ULTRA-TURRAX® Homogenizer (IKA, Staufen im Breisgau, Germany). Protein concentrations were determined using a Qubit 2.0 Fluorometer (Thermofisher, Waltham, Massachusetts, USA).

Protein extracts were analyzed at the Bioanalytical Mass Spectrometry Facility at the Mark Wainwright Analytical Centre (UNSW, Australia) using label-free quantification mass spectrometry using standard protocol. Briefly, samples were digested with Trypsin (MS Grade, Thermofisher) and separated by nanoLC using an Ultimate nanoRSLC UPLC and autosampler system (Dionex, Amsterdam, Netherlands). Samples (2.5 μl) were concentrated and desalted with a micro C18 precolumn with H_2_O:CH_3_CN (98:2, 0.1% TFA) at 15 μl/min and a fritless nano column (75 μm × 15 cm) containing C18-AQ media (Dr Maisch, Ammerbuch-Entringen Germany). Peptides were eluted using a linear gradient of H_2_O:CH_3_CN (98:2, 0.1% formic acid) to H_2_O:CH_3_CN (64:36, 0.1% formic acid) at 200 nl/min over 60 min. 2000 V was applied to low volume titanium union and the tip positioned ~ 0.5 cm from the heated capillary (T = 275 °C) of an Orbitrap Fusion Lumos (Thermo Electron, Bremen, Germany) mass spectrometer. Positive ions were generated by electrospray and the Fusion Lumos operated in data dependent acquisition mode (DDA).

A survey scan m/z 350–1750 was acquired in the orbitrap (resolution = 120,000 at m/z 200, with an accumulation target value of 400,000 ions) and lockmass enabled (m/z 445.12003). Data-dependent tandem MS analysis was performed using a top-speed approach (cycle time of 2 s). MS2 spectra were fragmented by HCD (NCE = 30) activation mode and the ion-trap was selected as the mass analyzer. The intensity threshold for fragmentation was set to 25,000. A dynamic exclusion of 20 s was applied with a mass tolerance of 10 ppm.

Peak lists were generated using Mascot Daemon/Mascot Distiller (Matrix Science, London, England) and imported into the database search program Mascot (version 2.6.0, Matrix Science). Search parameters were: Precursor tolerance 4 ppm and product ion tolerances ±0.5 Da; Met (O) carboxyamidomethyl-Cys specified as variable modification, enzyme specificity was trypsin, with 1 missed cleavage possible. Peaks were searched against the reference genome of the central bearded dragon [17] and a non-redundant protein database from NCBI (Jan 2015).

Raw peak data were imported into Scaffold (Matrix Science, London, England) and analysed accordingly with default settings. Normalized peak lists were imported into R (3.4.2) [103] for analysis. Proteins were excluded if there was an average of less than three spectral counts across the biological replicates in both conditions. Proteins were considered differentially expressed if the standard error of the mean spectral counts of each condition (i.e. hibernation vs. post-arousal) did not overlap.

## Additional files

Additional file 1: **Table S1.** Differential gene expression of RNA sequencing. Full list of differentially expressed genes with FDR < 0.05 for brain, heart, skeletal muscle, and common genes as outputted from EdgeR. Log_2_ fold change is relative to hibernation (i.e. > 1 Log_2_FC is higher expression during hibernation). (XLSX 1302 kb)

Additional file 2: **Table S2.** Gene ontology enrichment analysis data of differentially expressed genes. Full list of enriched biological pathway gene ontologies in upregulated and downregulated gene datasets in brain, heart, skeletal muscle, and common genes as outputted fromx GOrilla. (XLSX 140 kb)

Additional file 3: **Table S3.** Protein expression data. Full list of all proteins identified with label-free mass spectrometry by Scaffold. (XLSX 82 kb)

Additional file 4: **Table S4.** Gene Set Enrichment Analysis (GSEA) results of RNA sequencing data. GSEA results of RNA sequencing data, containing Biocarta, KEGG, Reactome, and Biological Pathway results. (XLSX 835 kb)

## Abbreviations

ATAC: Ada2a-containing
BMP: Bone morphogenetic protein
CPM: Counts per million
CT: Circadian time
FDR: False discovery rate
GO: Gene Ontology
GSEA: Gene set enrichment analysis
HDAC: Histone deacetylase
KEGG: Kyoto Encyclopedia of Genes and Genomes
MAPT: Microtubule associated protein tau
miRNA: microRNA
mRNA-seq: mRNA sequencing
MSTN: Myostatin
NMDAR: N-methyl-D-aspartate receptor
RISC: RNA-induced silencing complex
SUMO: Small ubiquitin-like modifiers
TCA: Tricarboxylic acid
TGF-β: Transforming growth factor beta-receptor
ZT: Zeitgeber time
